# Is there a relationship between malocclusion and *bullying*? A systematic review

**DOI:** 10.1186/s40510-020-00323-7

**Published:** 2020-09-01

**Authors:** Sylvia Karla P. C. Tristão, Marcela B. Magno, Andréa Vaz Braga Pintor, Ilana F. O. Christovam, Daniele Masterson T. P. Ferreira, Lucianne Cople Maia, Ivete Pomarico Ribeiro de Souza

**Affiliations:** 1 Department of Pediatric Dentistry and Orthodontics, Universidade Federal do Rio de Janeiro, R. Prof. Rodolpho Paulo Rocco, 325., Rio de Janeiro, RJ 21941-617 Brazil; 2 Library of Health Science Center, Universidade Federal do Rio de Janeiro, Rio de Janeiro, RJ Brazil

**Keywords:** Malocclusion, Bullying, Child, Adolescent

## Abstract

**Background:**

Malocclusion is a highly prevalent public health problem, and several studies have shown its negative correlation with quality of life, self-esteem, and social perceptions. However, its association with bullying is still controversial.

**Objectives:**

To evaluate the relationship between malocclusion and bullying in children and adolescents.

**Search methods:**

The databases used for the electronic researches were PubMed, Scopus, Lilacs/BBO, Web of Science, and Cochrane Library. Grey literature was reviewed through Open Grey literature with no language or date restrictions. Selection criteria, based on the PECO strategy, were considered eligible observational studies that included schoolchildren or adolescents (P) with malocclusion (E), compared to those with normal occlusion (C), in which the relationship between malocclusion and bullying was determined (O).

**Data collection and analysis:**

Risk of bias evaluation was made for the qualitative synthesis by the Fowkes and Fulton criteria. Data regarding the age of participants and types of malocclusion and of bullying were extracted among other reported data. The quality of the evidence analyzed was evaluated through the GRADE approach.

**Results:**

From 2744 articles identified in databases, nine met the eligibility criteria and were included in present systematic review, of which two studies were judged with methodological soundness. The quality of the evidence was classified as very low due to very serious problems for “risk of bias” and “other considerations” and serious problems of “indirectness”. The age of participants ranged from 9 to 34 years considering a cohort study, with a bullying recalling perspective. Malocclusion was both evaluated by researchers and self-reported by participants addressing dentofacial characteristics mostly related to the incisors relationship. All studies evaluated the verbal type of bullying, while 3 also considered physical type. Both types were reported as related to malocclusion, although the results showed that extreme maxillary overjet (> 4 mm, > 6 mm, > 9 mm), extreme deep overbite, and having space between anterior teeth or missing teeth were the types of malocclusion with the strongest relations to bullying.

**Conclusions:**

With very low certainty of evidence, the results of this systematic review suggest that conspicuous extreme malocclusion may be related to the occurrence of bullying among children and adolescents.

## Introduction

Bullying is defined as a practice of aggressive behavior or intentional harm to which an individual is repeatedly exposed in a relationship characterized by an imbalance of power [1]. Bullying may be direct, when it involves physical or verbal aggression, or indirect, when it involves manipulation of social relationships with gossip or exclusion, which is the most frequent direct form, consisting of verbal abuse associated with derogatory nicknames [2, 3]. Bullying has been observed for a long time, and its prevalence varies depending on location and age and may be as high as 88% [4], turning into a global concern [5].

Physical characteristics and esthetic patterns are remarkably meaningful in society, and such patterns are observed both in childhood and adolescence, periods during which they are more intense, because insertion and acceptance in the social group take on a central role [6]. The factors that trigger bullying often include social, religious, and physical characteristics that distinguish the victim from other members of the group [7]. The general physical characteristics most commonly observed for nicknames are weight, height, and facial appearance [8]. Given that the dentofacial region significantly contributes to general facial appearance and a harmonious smile plays an important role in facial beauty [9], it seems reasonable to assume that misaligned teeth and/or lack of harmony between maxillary bones and the mandible, or malocclusion, may be associated with bullying.

Malocclusion is a highly prevalent public health problem [10, 11] and several studies have shown its negative correlation with quality of life [12], self-esteem [13], and social perceptions [14]. However, its relationship with bullying is still controversial. Some authors have reported a higher prevalence of bullying among children and adolescents with malocclusion and dentofacial features [15, 16], whereas Agel et al. [17] have not found any evident relationship between the frequency of bullying at school and the increased overjet or lip incompetence. To fill this gap in knowledge, this study systemically reviews the literature aiming to answer the question: “Is there a relationship between malocclusion and bullying in schoolchildren or adolescents?”

## Materials and methods

### Study protocol

This systematic review is registered in the PROSPERO database (no. CRD42016042211), which was built following the Preferred Reporting Items for Systematic Reviews and Meta-Analyses (PRISMA) guidelines [18].

### Search strategies, study selection, and eligibility criteria

The electronic search was made up to January 2020 using the PubMed, Scopus, Lilacs/BBO, Web of Science, Cochrane Library databases, and Open Grey. The search strategy included MeSH terms and keywords related to the aim of this review, with no restrictions on language or date, and adapted to each database according to their syntaxes rules (Table 1). The whole process was overseen by a librarian (D.M.T.P.F.). A manual search was carried out in the reference lists of the articles selected for the systematic review in order to detect relevant publications possibly missed in the database searches. Articles retrieved from more than one database were computed only once.

**Table 1:** Search strategies

**Pubmed**	((malocclusion[MeSH Terms] OR malocclusion*[Title/Abstract] OR dentistry[MeSH Terms] OR Class I[Title/Abstract] OR Class II[Title/Abstract] OR Class III[Title/Abstract] OR dental esthetic*[Title/Abstract] OR overjet[Title/Abstract] OR overbite[Title/Abstract] OR protrusion[Title/Abstract] OR retrognathism mandibular[Title/Abstract] OR maxillofacial[Title/Abstract] OR dental occlusion[Title/Abstract] OR tooth[Title/Abstract] OR teeth[Title/Abstract] OR orthodont*[Title/Abstract] OR incompetent lips[Title/Abstract])) AND ((aggressions[MeSH Terms] OR aggression*[Title/Abstract] OR *bullying*[MeSH Terms] OR *bullying*[Title/Abstract] OR bullied[Title/Abstract] OR Social Marginalization[MeSH Terms] OR Social marginalization[Title/Abstract] OR Social Isolation[MeSH Terms] OR Stress Disorders, Post-Traumatic[MeSH Terms] OR Post-Traumatic Stress Disorder[Title/Abstract] OR Phobic Disorders[MeSH Terms] OR discrimination social[Title/Abstract] OR harassment[Title/Abstract] OR intimidation[Title/Abstract] OR Social Phobia[Title/Abstract] OR Social Isolations[Title/Abstract] OR teas*[Title/Abstract] OR nickname[Title/Abstract]))
**Scopus**	(malocclusion* OR dentistry OR “Class I” OR “Class II” OR “Class III” OR “dental esthetic” OR “dental esthetics” OR overjet OR overbite OR protrusion OR “retrognathism mandibular” OR maxillofacial OR “dental occlusion” OR tooth OR teeth OR orthodont* OR “incompetent lips”) AND (aggression* OR *bullying* OR bullied OR “Social Marginalization” OR “Social Isolation” OR “Post-Traumatic Stress Disorder” OR “Phobic Disorders” OR “discrimination social” OR harassment OR intimidation OR “Social Phobia” OR “Social Isolations” OR teas* OR nickname")
**Web of Science**	((malocclusion* OR dentistry OR “Class I” OR “Class II” OR “Class III” OR dental esthetic OR overjet OR overbite OR protrusion OR “retrognathism mandibular” OR maxillofacial OR “dentalocclusion” OR tooth OR teeth OR orthodont* OR “incompetent lips”) AND (aggression* OR *bullying* OR bullied OR “Social Marginalization” OR “Social Isolation” OR “Post-Traumatic Stress Disorder” OR “Phobic Disorders” OR “discrimination social” OR harassment OR intimidation OR “Social Phobia” OR “Social Isolation” OR teas* OR nickname*) )
**Lilacs /BBO**	(“Malocclusion, Angle Class I” OR “Mal Oclusão de Angle Classe I” OR “Malocclusion, Angle Class II” OR “Mal Oclusão de Angle Classe II” OR “Malocclusion, Angle Class III” OR “Mal Oclusão de Angle Classe III” OR malocclusion OR “Mal Oclusão” OR “esthetics, dental” OR “estética dentária” OR dentistry OR odontologia OR “tooth crowd” OR “Apinhamento dentário” OR crossbite OR “mordida cruzada” OR overjet OR sobressaliência OR overbite OR sobremordida OR “open bite” OR “mordida aberta” OR protrusion OR protrusão OR “retrognathic mandible” OR “mandíbula retrognata” OR underbite OR “mordida invertida” OR maxillofacial OR “Maxilo facial” OR “dental occlusion” OR “oclusão dentária” OR tooth OR dente OR teeth OR dentes OR dentition OR dentição OR orthodontic OR ortodôntico OR “aesthetic dental” OR “estética dental” OR “cosmetic dentistry” OR “estética dentária” OR “incompetent lips” OR “incompetência labial” OR “gummy smile” OR “sorriso gengival”) AND (Aggressions OR agressão OR *bullying* OR “assédio moral” OR “Social Marginalization” OR “marginalização social” OR “Social Isolation” OR “isolamento social” OR “Phobic Disorders” OR “transtornos fóbicos” OR bullied OR intimidado OR discrimination OR discriminação OR harassment OR assédio OR intimidation OR intimidação OR“ Post-Traumatic Stress Disorder” OR “Transtorno de estresse pós traumatico” OR “Social Phobia” OR “fobia social” OR “School Phobia” OR “fobia escolar” OR “Social Isolations” OR “isolamento social” OR “Social marginalization” OR “marginalização social” OR teas* OR nickname*)
**Open Grey**	(“Malocclusion, Angle Class I” OR “Maloclusão de Angle Classe I” OR “Malocclusion, Angle Class II” OR “Maloclusão de Angle Classe II” OR “Malocclusion, Angle Class III” OR “Maloclusão de Angle Classe III” OR malocclusion OR “Maloclusão” OR “esthetics, dental” OR “estética dentária” OR dentistry OR odontologia OR “tooth crowd” OR “Apinhamento dentário” OR crossbite OR “mordida cruzada” OR overjet OR sobressaliência OR overbite OR sobremordida OR “open bite” OR “mordida aberta” OR protrusion OR protrusão OR “retrognathic mandible” OR “mandíbula retrognata” OR underbite OR “mordida invertida” OR maxillofacial OR “Maxilo facial” OR “dental occlusion” OR “oclusão dentária” OR tooth OR dente OR teeth OR dentes OR dentition OR dentição OR orthodontic OR ortodôntico OR “aesthetic dental” OR “estética dental” OR “cosmetic dentistry” OR “estética dentária” OR “incompetent lips” OR “incompetência labial” OR “gummy smile” OR “sorriso gengival”) AND (Aggressions OR agressão OR *bullying* OR “assédio moral” OR “Social Marginalization” OR “marginalização social” OR “Social Isolation” OR “isolamento social” OR “Phobic Disorders” OR “transtornos fóbicos” OR bullied OR intimidado OR discrimination OR discriminação OR harassment OR assédio OR intimidation OR intimidação OR“ Post-Traumatic Stress Disorder” OR “Transtorno de estresse pós traumatico” OR “Social Phobia” OR “fobia social” OR “School Phobia” OR “fobia escolar” OR “Social Isolations” OR “isolamento social” OR “Social marginalization” OR “marginalização social” OR teas* OR nickname*)

After exclusion of duplicate articles, three reviewers (I.F.O.C., M.B.M., and S.K.P.C.T.) conducted an independent analysis of the list of titles and abstracts following the eligibility criteria. When the title and abstract did not provide enough information for the selection, the full article was read. If access to the full article was not possible, five attempts with authors and coauthors using different platforms, such as e-mail and social networks, were made with a week interval between the attempts. After the full article and the selected works were read, a decision was made as to whether the study should or should not be included. To complement the review, a manual search was made in the list of references of the selected works so as to find other relevant articles. Disagreements were resolved by a fourth reviewer (L.C.M.) after a consensus meeting. Following the complete reading of the selected articles, two reviewers (M.B.M. and S.K.P.C.T.) assessed the risk of bias of all studies.

The eligibility criteria were based on the PECO strategy [18]. In this sense, observational studies that included schoolchildren or adolescents (P) with malocclusion (E), compared to those with normal occlusion (C), in which the relationship between malocclusion and bullying was determined (O), were considered eligible for inclusion in this systematic review. In addition, the following exclusion criteria were established: reviews of the literature, letters to the editor, case reports, studies with other outcomes, studies that did not report on the relationship between bullying and malocclusion on schoolchildren or adolescents, or that did not provide a normal occlusion control group were excluded.

### Data extraction

The following data were extracted from the selected studies, as described in the table, by two reviewers (S.K.P.C.T. and M.B.M): information on the studies (author, year of publication, country of origin, and design), information on participants (total number of participants and age range), information on the methodology (terms related to bullying, instruments used to assess bullying, instruments used to assess malocclusion, their evaluation, and statistical analysis), information on the results (prevalence of malocclusion and its relationship with bullying), and conclusions.

### Risk of bias

The qualification of the studies and assessment of the risk of bias were made by two independent reviewers (S.K.P.C.T. and I.F.O.C.), in compliance with the guidelines established by Fowkes and Fulton [19]. This analytical model applies to the classification of cross-sectional, cohort, and case-control studies. The checklist included questions about the study model, the sample, the control group, the quality of the measures and of the outcomes, compliance, and possible distortions. The risk of bias of each article was classified as (++) major, (+) minor, (0) no bias, and (NA) not applicable, i.e., when the question did not apply to the methodology applied in studies included in the systematic review. Risk of bias classification criteria is described in Supplemental Table (ST1).

After a thorough appraisal of the methods and results, the criteria used to define an article as presenting low risk of bias or according to Fowkes and Fulton [19], considered as “quite sound,” were based on the answers to the recommended summary questions. Therefore, for the final assessment of methodological quality of the studies, three summary questions were asked regarding the following: (1) bias (Are the study outcomes incorrectly biased towards a given direction?), (2) confounding (Is there any influence that could lead to confounding or distortions?), (3) chance (Is it likely that the outcomes occurred by chance?). The answers to each of these questions could be either yes or no. Studies in which answers were “No” to all questions had a positive classification as compared to the other studies (studies with at least 2 “No’s”) and were considered to be methodologically quite sound.

### Quality of evidence

GRADE (Grading of Recommendations Assessment, Development, and Evaluation) [20] was used to analyze the quality of evidence. GRADE is a grading system for quality of evidence and for strength of health recommendations. When serious or extremely serious issues related to risk of bias, inconsistency, indirect evidence, inaccuracy, and publication bias are observed, the quality or certainty of evidence decreases by one or two points and could be classified as low or very low. Conversely, if there is large or very large magnitude of an effect that means a dose-response was observed. Also, if the effect of all plausible confounding factors is minimized or suggests a spurious effect, the quality of evidence tends to increase by two points and could be classified as moderate or strong. In this respect, the quality of evidence in GRADE may range between very low and strong.

For the criterion “risk of bias,” it was considered a “not serious” problem if all included studies presented methodological soundness and a very serious problem if one or more studies presented some type of methodological problem. For the “inconsistency” criterion, it was considered a very serious problem if the studies included in the systematic review presented a large variation in the effect estimates between studies.

The external validity was assessed whether the pooled results partially addressed the issue of interest for revision in the population (children and adolescents, exposure to only one type of malocclusion) or if the assessment to malocclusion occurred exclusively through a self-questionnaire (without clinical evaluation). If there was a limitation in one of these criteria, the problem was judged to be “serious”; if there was a problem in both criteria, the problem was judged to be “very serious.” In the analysis of “imprecision,” it was considered a serious problem if (1) the total number of patients evaluated was less than 300.

The criterion “publication bias” was judged to be “undetected” since the search was done in white and gray databases, with no date or language limitation. The criterion “dose-response” does not apply to the studies included in this systematic review and was classified in such a way, as not to modify the final classification of the evidence.

Grade approach was performed by two independent reviewers (S.K.P.C.T. and M.B.M.) who conducted this evaluation to determine the certainty of evidence of relationship between malocclusion and bullying.

## Results

**Fig. 1:**
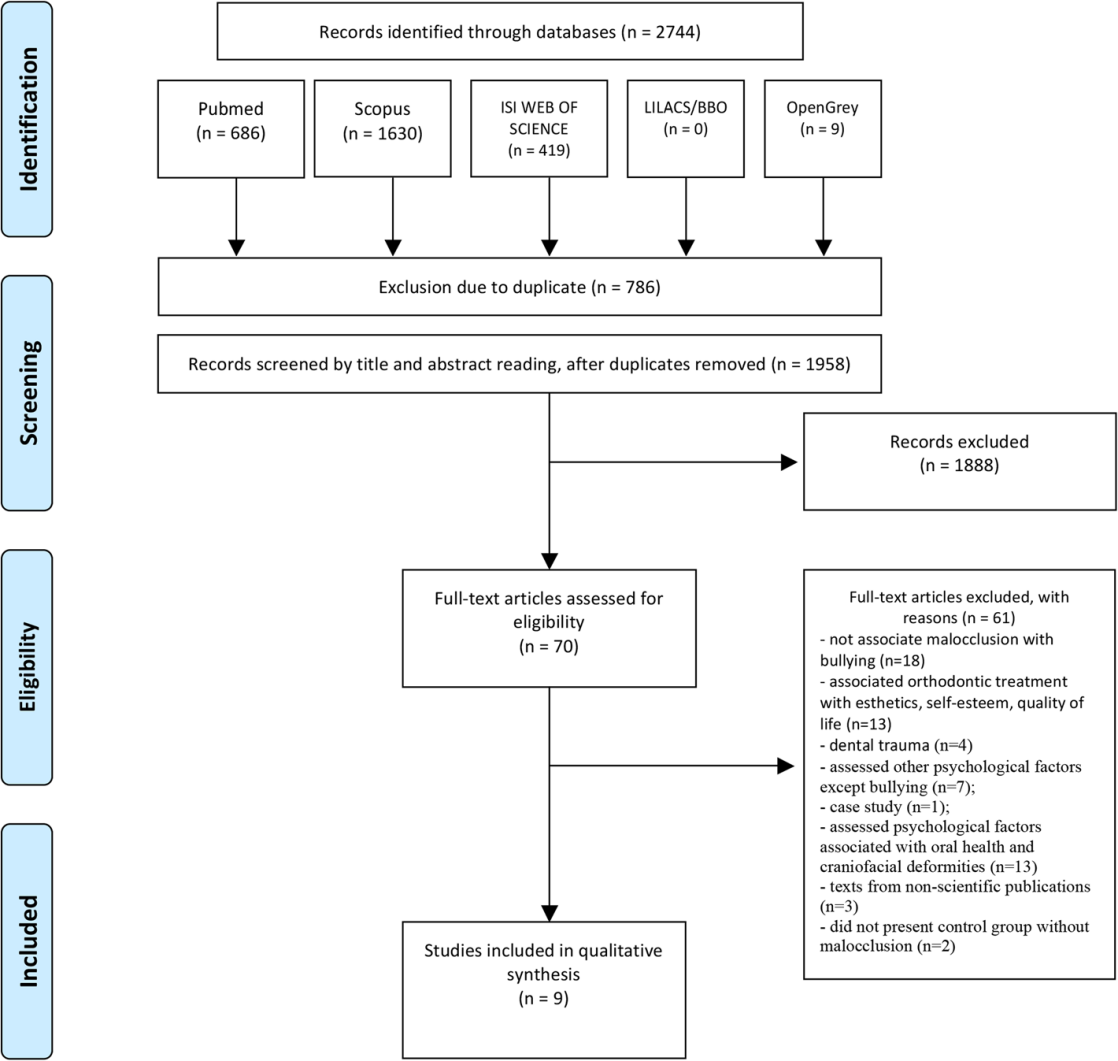
PRISMA flow diagram

The study selection results are presented in Fig. 1. A total of 2744 studies were retrieved, and 1958 remained after removing duplicates of different searched databases. After the titles and abstracts were read, 70 full articles were assessed for eligibility. Of the 70 full articles, 61 were excluded for the following reasons: they did not associate malocclusion with bullying (*n*=18); they associated orthodontic treatment with esthetics, self-esteem, quality of life (*n*=13), and dental trauma (*n*=4); they assessed other psychological factors except bullying (*n*=7); case study (*n*=1); they assessed psychological factors associated with oral health and craniofacial deformities (*n*=13); they consisted of texts from non-scientific publications (*n*=3); and did not present control group without malocclusion (*n*=2) (Supplemental Table ST2). Finally, nine articles were included in the present systematic review, and their risk of bias and quality of evidence were then assessed.

### Characteristics of the studies included in the systematic review

The characteristics of the studies included in this review are shown in Tables 2 and 3. Eight studies [15–17, 21, 22, 24–26] had a cross-sectional design and, one was a cohort study [23]. The studies were conducted in the UK, Denmark, Tanzania, Jordan, Nigeria, and Peru. The number of participants ranged from 147 to 920, whereas aged ranged from 9 to 18 in the cross-sectional studies [15–17, 21, 22, 24–26] and from 13 to 34 years in a cohort study with a bullying recalling perspective [23]. Six studies used the term “bullying” [15–17, 24–26], four used the term “tease” [21–24], and one study also used the terms “nicknames” and “harassment” [22] to refer to bullying. Regarding the assessment of the identification and prevalence of bullying, two studies used the questionnaire developed by Olweus Bully/Victim [16] or adaptations thereof [17]; one study used a questionnaire proposed by Ng’ang’a et al. [24]; another one employed the global negative self-evaluation (GSE) scale [21]; three studies used the questionnaire developed by Shaw et al. [22] adapted by Al-Bitar et al. [15, 25, 26]; whereas one assessed the prevalence of bullying using a single question (“Did your schoolmates tease you about the appearance of your teeth or jaws?”) [23].

**Table 2:** Data extracted from the included studies

Author, year, country	Study design	Total number of participants	Age range (years)	Terms used to refer to bullying/Type of bullying	Instrument used to assess bullying	Instrument used to assess malocclusion	Malocclusions evaluated/self-reported	Statistical analysis	Malocclusion outcomes	Association between malocclusion and bullying	Conclusions
Shaw et al, 1980 [22], UK	Cross-sectional	531	9–13	Nicknames, teasing, harassment/ verbal and physical	Interview questionnaire	Questionnaire about nicknames, teasing, harassment and physical characteristics	Not specified	Descriptive analysis and Chi-square	Not reported	66% were teased about their physical characteristics. 7% of total sample were teased about their teeth, of which 51% due to incisal prominence, 8% due to crowding. Children teased about teeth were twice likely to suffer harassment (55%) than those were not (26%) (*p* < 0.001)	Malocclusion was significantly related with bullying.
Helm et al, 1985 [23], Denmark	Cohort	758	First phase 13–19 Second phase 28–34	Teasing/verbal	Fifth question of an own questionnaire: “Did your schoolmates tease you about the appearance of your teeth or jaws?”	Instrument used previously by Bjork et al. 1964	Max Ovj > 6 mm, Max Ovj > 9 mm, Mand Ovj, DB > 5 mm, DB > 7 mm, AOB, CrsB, Scissor bite, Crw Max inc, Crw Mand inc, Spacing Max inc.	Chi-square and Fisher exacts test	80% (*n* = 606) presented malocclusion	9% of adults with malocclusion suffered teasing during adolescence, and 1.3% of adults without malocclusion suffered teasing during adolescence (*p* < 0.001).	Extreme maxillary overjet, extreme deep bite, and space anomaly malocclusions were significantly related with teasing.
Rwakatema et al, 2006 [24], Tanzania	Cross-sectional	298	12–15	Teased and bullying/ verbal	Ng’ang’a et al. questionnaire. Question: “Do your schoolmates tease you about the appearance of your teeth or jaws?”	Ng’ang’a et al. questionnaire. Question: “Do you generally observe that your teeth are not appropriately aligned in your mouth?”	Self-reported: teeth alignment	Chi-square	56% respondents thought their teeth were properly aligned and 69% (*n* = 205) related that they need orthodontic treatment.	Bullying or teasing was not significantly related to teeth alignment (*p* = 0.093, 0 > 0.05)	Malocclusion was not significantly related with teasing or bullying.
Badran et al., 2010 [21], Jordan	Cross-sectional	400	14–16	Teasing/ verbal	Global negative self-evaluation (GSE) scale	IOTN (AC and DHC), self-perceived AC, perceived need for orthodontic treatment	Not specified	Spearman correlation coefficient	82% (*n* = 338) presented little, borderline, or definite need for orthodontic treatment.	Teasing about teeth was correlated with GSE scale (0.272), with students’ high AC score (0.213) and with perceived treatment need (0.354).	Malocclusion was significantly related with teasing.
Seehra et al., 2011, [16] UK	Cross-sectional	336	10–14	Bullying/verbal and physical	Olweus Bully/Victim questionnaire	IOTN (AC and DHC)	Incisor relationship, DHC and AC component of IOTN, skeletal pattern, FMPA, LFH, increased Ovj and over bite	Chi-square and Fisher exacts test	96% (*n* = 324) presented little, borderline, or definite need for orthodontic treatment.	Bullying was significantly related to Class II division 1 incisor relationship (*p* = 0.041), increased overbite (*p* = 0.023), and increased overjet (> 4 mm)(*p* = 0.001) and high need for OT by AC component of IOTN (*p* = 0.0014)	Malocclusion was significantly related with bullying, principally to the AC component of IOTN.
Al-Bitar et al., 2013 [15], Jordan	Cross-sectional	920	11–12	Bullying/verbal	Self-questionnaire with component for personal experience of bullying	Self-questionnaire with component for general physical characteristics and dentofacial features	Self-reported: AOB; spacing between teeth or missing teeth; Crw of teeth; gummy smile; prominent anterior teeth; prominent Mand anterior teeth; retrognathic Mand; incompetent lip coverage; prognathic Mand.	Descriptive statements with total frequency	Not reported	73% of adolescents reported they were victims of bullying due to dentofacial features. Teeth (50%), lips (14%), and chin (9%). Space between teeth or missing teeth was the most targeted feature.	Malocclusion was related with bullying.
Agel et al., 2014, [17], East UK	Cross-sectional	728	15–16	Bullying/verbal and physical	Six items derived from the revised Olweus Bully/Victim questionnaire	WHO oral clinical exam methodology	Ovj and lip coverage	Chi-square	1.51% of the adolescents presented Ovj > 6 mm and 0.41% presented inadequate lip coverage.	*Bullying* was not significantly related to Ovj > 6 mm or inadequate lip coverage (*p* > 0.05).	Malocclusion addressed by increased overjet was not significantly related with bullying.
Chikaod et al., 2017 [25], Nigeria	Cross-sectional	835	12–17	Bullying/verbal	Self-administered questionnaire modified from Al-Bitar 2013.	Self-administered questionnaire modified from Al-Bitar 2013.	Self-reported: space incisor, prominent Ovj, incompetent lip coverage, gummy smile, diastema or missing teeth, AOB, prognathic Mand, retrognatic Mand, prominent Mand anterior teeth, Crw.	Descriptive statements with total frequency	Not reported	51.9% of adolescents reported they were victims of bullying due to dentofacial features. Teeth (24.3%), chin (15.3%), and lips (12.3%). Space between teeth or missing teeth (12.2%) was the most common dentofacial feature identified as target for bullying.	Bullies frequently target to general dentofacial appearance (malocclusion).
Julca-Ching et al. 2019 [26], Peru	Cross-sectional	147	12–18	Bullying/verbal	Self-administered questionnaire modified from Al-Bitar 2013.	DAI	Not specified	Kruskal-Wallis	87.76% of adolescents presented malocclusion	*Bullying* was not significantly related to malocclusion (*p* > 0.05).	Malocclusion was not significantly related with bullying.

*IOTN* Index of Orthodontic Treatment Needs, *AC* esthetic component, *DHC* dental health component, *OT* orthodontic treatment, *Max Ovj* maxillary overjet, *Mand ovj* mandibular overjet, *DB* deep bite, *CrsB* crossbite, *AOB* anterior open bite, *Crw Max Inc* crowded maxillary incisor, *Crw Mand Inc* crowded mandibular incisor, *FMPA* Frankfort-Mandibular Plane Angle, *LFH* lower facial height

**Table 3:** Main dentofacial characteristics or types of malocclusions of interest reported in the included studies

Author, year, country	Chin	Lips, Lip incompetence or coverage	Teeth	Space between teeth, missing teeth	Crowding	Deep bite, > 5 mm, > 7 mm	Prominent anterior teeth, incisal prominence, incisor relationship	Maxillary overjet, > 4 mm, > 6 mm, > 9 mm	Cross bite, scissors bite	Retrognathic mandible	Prominent mandibular anterior teeth	Prognathic mandible	Anterior open bite	Gummy smile	Cephalometric measurements
Shaw et al, 1980 [22], UK	_	+, _	+	_	+	_, _, _	+, +, +	+, _, _, _	_, _	_	_	_	_	_	_
Helm et al, 1985 [23],Denmark	_	_, _	+	+, _	+	+, +, +	+, +, +	+, _, +, +	+, +	_	+	_	+	_	_
Rwakatema et al, 2006 [24], Tanzania	_	_	+	_	_	_, _, _	_	_	_	_	_	_	_	_	_
Badran et al., 2010 [21], Jordan	_	_, _	+	+, +	+	+, _, _	+, +, +	+, _, _, _	+, _	_	+	_	+	_	_
Seehra et al., 2011 [16], UK	+	+, +	+	+, +	+	+, _, _	+ ,+, +	+, +, +, +	+, +	+	+	+	+	_	+
Al-Bitar et al. 2013 [15], Jordan	+	+, +	+	+, +	+	+, _, _	+,+,+	+, _, _, _	_, _	+	+	+	+	+	_
Agel et al., 2014 [17], East UK	_	+, +	+	_	_	_	+,+,+	+, _, +, +	_	_	_	_	_	_	_
Chikaod et al., 2017 [25], Nigeria	+	+, +	+	+, +	+	+, _, _	+,+,+	+, _, _, _	_, _	+	+	+	+	+	_
Julca-Ching et al. 2019 [26], Peru	_	_, _	+	+, +	_	_	+,+,+	+, _, _, _	_, _	_	_	_	+	_	_

+ Reported, _ not reported

The assessment of malocclusion was evaluated by researchers [16, 17, 21, 23] and/or self-reported by participants [15, 16, 21, 22, 24–26]. Distinct methodologies were used by the researchers; two studies used the Index of Orthodontic Treatment Need (IOTN) criteria [16, 21],one study used the oral clinical exam methodology of the World Health Organization to evaluate overjet [17], one study used the Dental Aesthetic Index [26], and one [23] employed the method developed by Bjorn, Krebs and Solow. On the question of self-administered questionnaires applied in the presence of researchers, three [15, 25, 26] studies used versions of the questionnaire of Shaw et al. [22] that included questions about general physical and dentofacial characteristics [15, 25, 26], and one study used the question “Do you generally observe that your teeth are not appropriately aligned in your mouth?” [24]; Shaw et al. [22] assessed the malocclusion by an interview questionnaire about physical characteristics, not detailed.

Based on the outcomes of the studies included in the present systematic review, prevalence of malocclusion/need for treatment was high, ranging from 56 [24] to 96% [16]. Most studies [15, 16, 21–23, 25] concluded that malocclusion is related with bullying, while Agel et al. [17] and Rwakatema et al. [24] and Julca-Ching et al. [26] concluded that bullying is not related with malocclusion. Some variables influenced these outcomes, such as the type of malocclusion and the evaluated IOTN component. According to Sheera et al. [16], the aesthetic component (AC) is significantly more associated with bullying than are the dental health component (DHC), overjet, and overbite.

Among the nine studies included in the systematic review, five [16, 22, 23, 25, 26] reported no difference between gender and regarding bullying, three [17, 21, 24] did not provide any information about it, and only one study [15] reported that boys experience more bullying than do girls.

### Risk of bias

Table 4 describes the assessment of the risk of bias of the nine studies included in the systematic review, classified according to Fowkes and Fulton [19].

**Table 4:** Results of methodological quality assessment of included studies, according to Fowkes and Fulton criteria

Guideline	Checklist		Shaw et al., 1980	Helm et al., 1985	Rwakatema et al., 2006	Badran et al., 2010	Seehra et al., 2011	Al-Bitar et al., 2013	Agel et al., 2014	Chikaoki et al., 2017	Julca-Ching et al. 2019
Study design appropriate to objective?	Objective	Common design	0	0	0	0	0	0	0	0	0
	Prevalence	Cross-sectional	NA	NA	NA	NA	NA	NA	NA	NA	NA
	Prognosis	Cohort	NA	NA	NA	NA	NA	NA	NA	NA	NA
	Treatment	Controlled trial	NA	NA	NA	NA	NA	NA	NA	NA	NA
	Cause	Cohort, case-control, cross-sectional	0	0	0	0	0	0	0	0	0
Completeness?	Compliance		NA	+	NA	NA	NA	NA	NA	NA	NA
	Dropouts		0	0	NA	NA	NA	NA	NA	NA	NA
	Deaths		NA	NA	NA	NA	NA	NA	NA	NA	NA
	Missing data		0	0	0	0	0	0	0	0	0
Distorting influences?	Extraneous treatments		NA	NA	NA	NA	NA	NA	NA	NA	NA
	Contamination		NA	NA	NA	NA	NA	NA	NA	NA	NA
	Changes over time		NA	NA	NA	NA	NA	NA	NA	NA	NA
	Confounding factors		0	++	++	0	0	0	0	++	++
	Distortion reduced by analysis		++	0	++	0	0	0	++	++	++
Summary questions	Bias—Are the results erroneously biased in a certain direction?		YES	YES	YES	NO	NO	NO	NO	YES	YES
	Confounding—Are there any serious confounding or other distorting influences?		YES	NO	YES	NO	NO	YES	YES	YES	YES
	Chance—Is it likely that the results occurred by chance?		YES	NO	NO	NO	NO	NO	NO	NO	NO

Seven studies [15–17, 21–23, 26] did not use any type of randomization, but authors of the present systematic review judged that this could not influence outcome evaluation and were classified as (+) for “sampling method.” Three studies [17, 22, 23] adopted a representative sample, and other three [24–26] did not mention such sample size calculation or representative sample, and were classified as minor and major problem, respectively.

In five studies [17, 21, 22, 24, 25], the inclusion criteria were not clearly established but could be identified in the text, been classified as (+) in “definition of controls.” Five studies [15, 17, 22, 24, 26] mentioned that case and control groups were not matching or did not report about matching, been classified with (++) for “matching/randomization” and “comparable characteristics.”

Six studies [15, 22–26] did not use a previously validated instrument to evaluate bullying and were classified as (++) in “validity,” while other two studies [17, 25] did not report about training or calibration of evaluators (++) for “quality control” parameter. All studies did not blind the evaluator; however, this could not influence outcomes, been classified as (+) for “blindness.”

Four studies [23–26] presented some confounding factors, and five studies did not present matching between case and control groups and did not perform statistical analysis to minimize this factor, been classified as (++) for “confounding factors” and “distortion reduced by analysis,” respectively.

Concerning “summary questions,” the outcomes of five studies [22–26] were possibly biased, and six studies [15, 17, 22, 24–26] revealed confounding factors or other distortions associated with the outcomes. It is analyzed that Shaw et al. [22] results could occur by chance. So, only two studies [16, 21] were considered to be methodologically sound, whereas the other seven were not [15, 17, 22–26].

The results of the studies were presented in different ways (correlation tests, based on mean and standard deviation, or frequencies), not allowing for quantitative analysis.

### Quality of evidence

Quality of evidence supporting the relationship between malocclusion and bullying of nine studies included in the present systematic review was very low according to GRADE (Table 5). This classification is due to very serious problems with risk of bias and inconsistency and serious problems related to external validity (indirectness).

**Table 5:** Evidence profile: relationship between malocclusion and bullying

Patient or population: Children and adolescents Exposure/intervention: malocclusion Comparison: with normal occlusion Outcome: *Bullying*			
No. of participants (studies)	Relative effect (95% CI)	Certainty	What happens
4.953 (9 observational studies)	Not estimable	⨁◯◯◯ VERY LOW ^a,b,c^	^a^Very serious problems for “Risk of bias”: Seven, of the nine, studies included in the present systematic review were judged with non-solid methodologies, with results erroneously biased in a certain direction and/or any serious confounding or other distorting influences. ^b^Serious problems for “Indirectness”: Shaw et al., Helm et al*.*, Chikaod et al., Al-Bitar et al*.*, and Rwakatema et al. applied self-report questionnaires to evaluate malocclusion, without clinical exam. ^C^Very serious problems in “Other considerations”: Seven, of the nine, studies could have any serious confounding or other distorting influences.

*CI* confidence interval

## Discussion

Systematic reviews have gained popularity in health-related research. They include an analysis of risk of bias of individual studies, which is necessary for an in-depth investigation into their methods and outcomes, verifying whether the methods were appropriate and whether the outcomes were sufficiently clear so that the objectives could be achieved [19]. Seven [15, 17, 22–26] out of the nine studies included in this systematic review failed to have sound methodologies, possibly interfering with the outcomes or biasing them somehow. The meta-analysis interprets data with a larger statistical power, but it does not detect when a study is not conducted properly [27]. Thus, some studies [16, 21] selected for the present review, albeit considered to be “methodologically sound,” presented heterogeneous statistical analyses that did not allow conducting the meta-analysis.

Bullying occurs when a child or adolescent is intimidated or victimized repeatedly over time by negative actions performed by one or more peers [1]. This review shows that there exists no terminological pattern in the articles, as some authors use the term teasing [21–23], whereas other authors use bullying [15, 24–26] or “nicknames” [15, 22]. Ross [28] posits that teasing should not always be identified as bullying, and also that teasing should be understood as a form of acceptance and dialogue among friends, where all of them are teased likewise, and thus teasing is not targeted at a specific person. On the other hand, Olweus believes a single but more serious case of harassment could be construed as bullying under some circumstances due to low-level nonverbal harassment, cruel teasing, social ostracism, malicious gossip, sexual harassment, ethnic insults, unreasonable territorial bans, destruction of someone’s property, extortion, and serious physical assault should be regarded as negative actions [29]. Therefore, with the aim of maximizing the search and retrieval of potentially eligible articles, the three terms related to the outcome (bullying, teasing, and nickname) were included in the search strategy of the present review and considered to be synonymous with bullying during the assessment of the studies.

Overall, this systematic review results suggest that children and adolescents with conspicuous malocclusion, such as extreme maxillary overjet (> 4 mm, > 6 mm, > 9 mm), extreme deep overbite, and having space between anterior teeth or missing teeth, would be bullied more often than those with normal occlusion. This might be associated with the fact that children with a normal dental appearance are considered to be prettier, smarter, and friendlier, whereas bad-looking ones are more prone to teasing and harassment [9], since it is impossible to conceal facial or dental deformities during social contact [30]. However, it is worth mentioning that in general, the majority of the population presents malocclusion, evidenced in the high prevalence observed in the included studies, in which the participants were evaluated by researchers [16, 21, 23, 26] and also in those that malocclusion assessment was additionally [16, 21] or exclusively self-reported [24]. Curiously, although the sample of Seehra et al. [16] was composed by children and adolescents referred to orthodontic assessment at a reference hospital, which could explain the high prevalence of orthodontic treatment need, high prevalence of malocclusion was likewise observed by Badran et al. [21] in a sample of schoolchildren randomly selected. In addition, the malocclusion was related to bullying in both studies, despite the distinct age groups [16, 21].

On the question of age, the samples included participants with different age groups. Some studies [15, 16, 22] included children and adolescents below 14 years, justifying that high prevalence of bullying was previously [1, 2] reported for this age group. Meanwhile, some included older participants [17, 21]; others included patients with age ranging from 12 to a maximum of 18 years old at the time of malocclusion and bullying assessment [24–26]. In particular, in the single cohort study [23], malocclusion was evaluated at the age range from 13 to 19 years old, while the occurrence of bullying in adolescence was evaluated in a recalling perspective at the age of 28 to 34 years that could possibly represent a memory bias. Shaw et al. [22] results pointed that slightly more young participants, at the age of 9–10 years (73%) old, suffered bullying than the older ones (65%; *p* < 0.05). This result corroborates with the literature, which points that, the prevalence of bullying in childhood and adolescence decreases with the increasing age [1, 2, 31]. Interestingly, in some studies [21, 23, 25] with older samples, bullying was likewise related to malocclusion.

Several studies demonstrate that malocclusion has negative effects on adolescents’ self-esteem [32, 33] and that self-esteem and esthetic self-perception are influenced by other people’s opinions [21]. In bullying victims, a combination of factors may act synergistically, associating bullying, malocclusion, self-esteem, and quality of life and causing a negative effect on their psychosocial status [16, 34]. Bullying among children and adolescents is a problem with severe and long-lasting effects [35]. Bullying victims may feel depressed, lonely, and anxious [31], and, quite often, they dread going to school, a place they find unpleasant and unsafe, which may affect their academic performance [36]. If an adult does not intervene through the adoption of anti-bullying strategies, these victims will probably continue to be repeatedly exposed to this violence, putting them at risk for continuous social rejection, with consequences into adolescence and adulthood [37].

Studies have shown that exposure to direct violence tends to decrease with age, as younger children suffer more bullying than do older ones [2, 31]. Regardless of age, bullying should not be regarded as normal in the construction of social relationships, since it indicates risk of acceptance of violent behavior, situations of vulnerability, and social maladjustments. Each case should be dealt with in a personalized fashion, as the psychological impact of bullying, irrespective of the cause, may be devastating to a child, with long-term effects [35].

Even though the literature describes that significantly more boys tend to be bullied [15, 38, 39], the present study does not corroborate this finding. This could be related to the fact that dental appearance seems to be a priority, regardless of sex [40]. However, it is important to highlight that four studies [16, 22, 23, 25] did not describe this relation. Hence, further studies on the association between malocclusion and bullying and on the differences in prevalence between boys and girls are needed.

While the present study reports that children and adolescents with malocclusion are more prone to bullying, it is not possible to affirm that bullying would cease, self-esteem would be improved, and social interactions would get better after malocclusion is treated. The association between orthodontic treatment and better self-esteem is still controversial [41, 42]. Moreover, children who are bullied tend to continue being victims even when physical or social changes occur, such as changing schools and wearing dental braces [41]. There are reports that children who suffered bullying due to malocclusion continue to be nicknamed for their oral conditions [41].

The present systematic review followed specific guidelines [18] respecting the strategies for the database search, without any restrictions on language, performing all procedures independently and in duplicate or triplicate, and taking all possible care to minimize bias to the extent possible.

However, some limitations became evident because of the experimental designs. Most of the included studies evaluated bullying through questionnaires not validated, besides the fact that in few studies the researchers evaluated malocclusion by orthodontic assessment tools criteria [16, 21, 26] and only one by a thorough orthodontic exam including images [16]. In addition, most of the studies were not considered methodologically sound, due to a sequence of absence/not reported methodological details. This contributed to the very low certainty of evidence, reinforcing the need of better methodologically conducted primary studies addressing the present issue. Meta-analyses were not performed as a result of scarcity and heterogeneity in quantitative data description.

Based on the findings of the present systematic review, the authors encourage further studies with good methodological quality, rigorous eligibility, and control group selection criteria, using instruments and measures that have been previously validated in the literature, training, and calibration of evaluators, and absence and/or statistical adjustments for confounding factors for investigation of the association between malocclusion and bullying in order to strengthen the evidences about this important issue.

## Conclusion

Despite the very low quality of evidence, the results of this systematic review suggest that conspicuous extreme malocclusion may be related to the occurrence of bullying among children and adolescents.

## Supplementary information

**Additional file 1: Supplemental Table ST1.** Criteria’s adopted to risk of bias classification.

**Additional file 2: Table ST2.** Full text evaluated and excluded from systematic review.

## Data Availability

The datasets used and analyzed during the current study are available from the corresponding author on reasonable request.

## Abbreviations

PRISMA: Preferred Reporting Items for Systematic Reviews and Meta-Analyses
PECO: Population-exposure-comparator-outcome
GRADE: Grading of Recommendations Assessment, Development and Evaluation
GSE: Global negative self-evaluation
IOTN: Index of Orthodontic Treatment Need
AC: Aesthetic component
DHC: Dental Health Component
WHO: World Health Organization
DAI: Dental Aesthetic Index
OT: Orthodontic treatment
Max Ovj: Maxillary overjet
Mand ovj: Mandibular overjet
DB: Deep bite
CrsB: Crossbite
AOB: Anterior open bite
Crw Max Inc: Crowded maxillary incisor
Crw Mand Inc: Crowded mandibular incisor
FMPA: Frankfort-mandibular plane angle
LFH: Lower facial height
NA: not applicable
++: Major
+: Minor
0: No bias

## References

[1] Olweus D (1994). Bullying at school: basic facts and effects of a school based intervention program. J Child Psychol Psychiatry.

[2] Boulton MJ, Underwood K (1992). Bully/victim problems among middle school children. Br J Educ Psychol.

[3] Solberg ME, Olweus D, Endresen IM (2007). Bullies and victims at school: are they the same pupils?. Br J Educ Psychol.

[4] Ometeso BA (2010). Bullying behaviour, its associated factors and psychological effects among secondary students in Nigeria. J Int Soc Res.

[5] Carney AG, Merrel KW (2001). Perspectives on understanding and preventing an international problem. School Psychol Int.

[6] Scheffel DL, Jeremias F, Fragelli CM, Dos Santos-Pinto LA, Hebling J, de Oliveira OBJr. Esthetic dental anomalies as motive for bullying in schoolchildren. Eur J Dentistry 2014;8(1):124-128. 10.4103/1305-7456.126266 PMC4054024 24966759

[7] Malta DC, Porto DL, Crespo CD, Silva MM, de Andrade SS, de Mello FC (2014). Bullying in brazilian school children: analysis of the national adolescent school-based health survey (pense 2012). Rev Bras Epidemiol.

[8] Kolawole KA, Otuyemi OD, Adeosun OD (2009). Nicknames and name calling among a population of Nigerian schoolchildren. Eur J Paediatr Dent.

[9] Shaw WC (1981). The influence of children’s dentofacial appearance on their social attractiveness as judged by peers and lay adults. Am J Orthodontics.

[10] Eslamipour F, Afshari Z, Najimi A (2018). Prevalence of malocclusion in permanent dentition of Iranian population: a review article. Iran J Public Health.

[11] Shen L, He F, Zhang C, Jiang H, Wang J (2018). Prevalence of malocclusion in primary dentition in Mainland, China, 1988-2017: a systematic review and meta-analysis. Scientific Reports.

[12] Dimberg L, Arnrup K, Bondemark L (2015). The impact of malocclusion on the quality of life among children and adolescents: a systematic review of quantitative studies. Eur J Orthod.

[13] Jung MH (2010). Evaluation of the effects of malocclusion and orthodontic treatment on self-esteem in an adolescent population. Am J Orthod Dentofacial Orthop.

[14] Pithon MM, Andrade D, Fernandes I, Mendes J, Nunes K, Michele L (2014). Influence of malocclusion on social perceptions of adolescents at public and private schools. Eur Arch Paediatr Dent.

[15] Al-Bitar ZB, Al-Omari IK, Sonbol HN, Al-Ahmad HT, Cunningham SJ (2013). Bullying among Jordanian schoolchildren, its effects on school performance, and the contribution of general physical and dentofacial features. Am J Orthod Dentofacial Orthop.

[16] Seehra J, Fleming PS, Newton T, DiBiase AT. Bullying in orthodontic patients and its relationship to malocclusion, self-esteem and oral health-related quality of life. J Orthod 2011;38(4):247-256 (a). 10.1179/14653121141641 22156180

[17] Agel M, Marcenes W, Stansfeld SA, Bernabe E (2014). School bullying and traumatic dental injuries in east London adolescents. Brit Dental J.

[18] Moher D, Liberati A, Tetzlaff J, Altman DG, Group P (2009). Preferred reporting items for systematic reviews and meta-analyses: the PRISMA statement. J Clin Epidemiol.

[19] Fowkes FG, Fulton PM (1991). Critical appraisal of published research: introductory guidelines. BMJ.

[20] Ryan R, Hill S. How to GRADE the quality of the evidence. Cochrane Consumers and Communication Group, available at http://cccrg.cochrane.org/author-resources. Version 3.0. 2016.

[21] Badran SA (2010). The effect of malocclusion and self-perceived aesthetics on the self-esteem of a sample of Jordanian adolescents. European Journal of Orthodontics.

[22] Shaw WC, Meek SC, Jones DS (1980). Nicknames, teasing, harassment and the salience of dental features among school children. Brit J Orthod.

[23] Helm S, Kreiborg , Solow, B. Psychosocial implications of malocclusion: a 15-year follow-up study in 30-year-old Danes. Am J Orthod 1985;87 (2):110-118. 10.1016/0002-9416(85)90020-x 3855604

[24] Rwakatema DS, Ng'ang'a PM, Kemoli AM (2006). Awareness and concern about malocclusion among 12-15 year-old children in MoshiTanzania. East Afr Med J.

[25] Chikaodi O, Abdulmanan Y, Emmanuel AT, Muhammad J, Mohammed, MA, Izegboya A. Bullying, its effects on attitude towards class attendance and the contribution of physical and dentofacial features among adolescents in northern Nigeria. Int J Adolesc Med Health 2017;31(2). 10.1515/ijamh-2016-0149 28731856

[26] Julca-Ching K, Carruitero MJ (2019). Impact of the need for orthodontic treatment on academic performance, self-esteem and bullying in schoolchildren. J Oral Res.

[27] Hinggs J, Green S (2011). Cochrane handbook for systematic reviews of interventions.

[28] Ross DM. Childhood bullying, teasing, and violence: what school personnel, other professionals, and parents can do. 2^nd^ ed. Alexandria, Va.: American Counseling Association; 2003. p. 293).

[29] Olweus D. Bullying at school: what we know and what we can do: United Kingdom, Blackwell Publishing; 1993. p. 152).

[30] Macgregor FC (1970). Social and psychological implications of dentofacial disfigurement. The Angle Orthodontist.

[31] Hawker DS, Boulton MJ (2000). Twenty years’ research on peer victimization and psychosocial maladjustment: a meta-analytic review of cross-sectional studies. J Child Psychol Psychiatry.

[32] Taibah SM, Al-Hummayani FM (2017). Effect of malocclusion on the self-esteem of adolescents. J Orthod Sci.

[33] Kaur P, Singh S, Mathur A, Makkar DK, Aggarwal VP, Batra M (2017). Impact of dental disorders and its influence on self esteem levels among adolescents. J Clin Diagnostic Res.

[34] Seehra J, Newton JT, DiBiase AT. Bullying in schoolchildren - its relationship to dental appearance and psychosocial implications: an update for gdps. Brit Dental J 2011;10(9):411-415 (b). 10.1038/sj.bdj.2011.339 21566605

[35] DiBiase AT, Sandler PJ (2001). Malocclusion, orthodontics and bullying. Dental update.

[36] Dorcas OF. Bullying in nigerian secondary schools: strategies for counseling intervention. Educ Res Rev 2015;10(4): 435–443.

[37] Olweus D. Victimisation by peers: antecedents and long-term outcomes. In Rubin K.H. (Ed.) Asendorf J.B.Social withdrawal, inhibition and shyness in childhood; 1993. p. 315–341.

[38] Craig W, Harel-Fisch Y, Fogel-Grinvald H, Dostaler S, Hetland J, Simons-Morton B (2009). A cross-national profile of bullying and victimization among adolescents in 40 countries. Int J Public Health.

[39] Serra-Negra JM, Paiva SM, Bendo CB, Fulgencio LB, Lage CF, Correa-Faria P (2015). Verbal school bullying and life satisfaction among brazilian adolescents: profiles of the aggressor and the victim. Comprehensive Psychiatry.

[40] Lerner RM, Karabenick SA, Stuart JL (1973). Relations among physical attractiveness, body attitudes, and self-concept in male and female college students. J Psychol.

[41] Seehra J, Newton JT, Dibiase AT (2013). Interceptive orthodontic treatment in bullied adolescents and its impact on self-esteem and oral-health-related quality of life. Eur J Orthod.

[42] Bernstein JY, Watson MW (1997). Children who are targets of bullying: a victim pattern. J Interpersonal Violence.

