# Supplementary material for: Is there a relationship between malocclusion and bullying? A systematic review
Source: Prog Orthod. 2020 Sep 1;21:26. doi: 10.1186/s40510-020-00323-7 (PMC7459069; doi:10.1186/s40510-020-00323-7)
Supplement: Supplementary file 1 — Additional file 1: Supplemental Table ST1. Criteria’s adopted to risk of bias classification. [file 40510_2020_323_MOESM1_ESM.docx]

**Supplemental Table ST1.** Criteria’s adopted to risk of bias classification.

| Study design appropriate to objective? | Prevalence - Cross-sectional | 0 – if the study, or part of study, included in the present systematic review evaluate some type of relationship between malocclusion and bullying had a cross-sectional design |
| --- | --- | --- |
|  | Prognosis - Cohort | NA as prognosis is not the objective of present systematic review. |
|  | Treatment - Controlled trial | NA as treatment is not the objective of present systematic review. |
|  | Cause - Cohort, case-control, cross-sectional | 0 - if the study, or part of study, included in the present systematic review evaluate some type of relationship / association / risk between malocclusion and bullying had a cross-sectional, case-control or cohort design, respectively. |
| Study sample representative? | Source of sample | 1. case and control groups were obtained from the same population source.   (+) not adopted*.  (++) case and control groups were obtained from different sources. |
|  | Sampling method | (0) study report low risk of bias sample randomization.  (+) did not use any type of randomization, but authors of the present systematic review judged that could not influenced in outcome evaluation.  (++) did not use any type of randomization, but authors of the present systematic review judged that could influenced in outcome evaluation. |
|  | Sample size | (0) sample size calculation was described.  (+) did not perform sample size calculation but had a representative sample.  (++) did not mention such sample size calculation or representative sample. |
|  | Inclusion/exclusion criteria | (0) studies that described such criteria  (+) not adopted*  (++) study that did not use inclusion and exclusion criteria. |
|  | Non-respondents | (0) study that reported non-respondents participants  (+) not adopted*  (++) study that not use this item. |
| Control group acceptable? | Definition of controls | (0) study described the inclusion criteria of the control group  (+) denoted those studies in which inclusion criteria were not clearly established but could be identified in the text  (++) denoted those studies in which inclusion criteria were not described and/or could be identified in the text |
|  | Source of controls | (0) case and control groups obtained from the same population  (+) not adopted*  (++) case and control groups obtained from different populations |
|  | Matching/randomization | (0) study mentioned that case and control groups were matching for sex and age  (+) study mentioned that case and control groups were matching for sex or age  (++) study mentioned that case and control groups were not matching or did not report about matching. |
|  | Comparable characteristics |  |
| Quality of measurements and outcomes? | Validity | (0) study that use a previously validated instruments.  (+) not adopted.  (++) study that use not valeted instruments. |
|  | Reproducibility | (0) study present sufficient information on the methodology that permit reproducibility  (+) not adopted*  (++) study whose description of the methodology was not enough to ensure their reproducibility |
|  | Blindness | (0) study in which the evaluator was blinded to the assessed factor (malocclusion)  (+) study that did not mention the use of blinding  (++) NA as the lack of blinding did not have a direct influence on the outcome |
|  | Quality control | (0) evaluator had been trained and calibrated for malocclusion assessment.  (+) evaluator had been trained but not calibrated for malocclusion assessment.  (++) there was no information in the study about training or calibration of evaluators.  NA was used for studies that assessed malocclusion by means of photographs or self-report. |
| Completeness? | Compliance | (0) if, in a cohort study, the number of dropouts was lower than 30%.  (+) if, in a cohort study, the number of dropouts was greater than 30%, and if a justification was given for authors.  (++) if, in a cohort study, had a dropout rate greater than 30% with no justification.  NA was used if the study had a cross-sectional or case-control design |
|  | Dropouts |  |
|  | Deaths | NA (question did not apply to study methodology) |
|  | Missing data | (0) study report all necessary data to respond your objective.  (+) not adopted+.  (++) study did not report some data important to respond your objective. |
| Distorting influences? | Extraneous treatments | NA (question did not apply to study methodology) |
|  | Contamination | NA (question did not apply to study methodology) |
|  | Changes over time | NA (question did not apply to study methodology) |
|  | Confounding factors | (0) no confounding factors were observed.  (+) not adopted*.  (++) some confounding factors were observed. |
|  | Distortion reduced by analysis | (0) studies that did not present matching between case and control groups, but perform statistical analysis to minimize this factor.  (+) not adopted*.  (++) studies that did not present matching between case and control groups and did not perform statistical analysis to minimize this factor. |

NA – Not applied.

*(+) was not adopted due author judged that the absence of criteria is a major problem.
