# Supplementary material for: Is there a relationship between malocclusion and bullying? A systematic review
Source: Prog Orthod. 2020 Sep 1;21:26. doi: 10.1186/s40510-020-00323-7 (PMC7459069; doi:10.1186/s40510-020-00323-7)
Supplement: Supplementary file 2 — Additional file 2: Table ST2. Full text evaluated and excluded from systematic review. [file 40510_2020_323_MOESM2_ESM.docx]

**Table ST2**. Full text evaluated and excluded from systematic review.

| Author | Reference | Year |
| --- | --- | --- |
| Macgregor FC | Social and psychological implications of dentofacial disfigurement. The Angle Orthodontist 1970; 40(3):231-233 | 1970 |
| Hitchin AD, Shuker ST. | Sociological aspects of maxillofacial injuries in the East of Scotland. Proc R Soc Med. 1973 Jul;66(7):699-700 | 1973 |
| Lerner RM, Karabenick SA, Stuart JL | Relations among physical attractiveness, body attitudes, and self-concept in male and female college students. The Journal of Psychology 1973; 85(1):119-129. | 1973 |
| Shaw WC, Meek SC, Jones DS | Nicknames, teasing, harassment and the salience of dental features among school children. British Journal of Orthodontics 1980;7(2):75-80 | 1980 |
| Shaw WC. | The influence of children's dentofacial appearance on their social attractiveness as judged by peers and lay adults. American journal of orthodontics 1981;79(4):399-415 | 1981 |
| Helm S, Kreiborg , Solow, B | Psychosocial implications of malocclusion: A 15-year follow-up study in 30-year-old danes. American Journal of Orthodontics 1985;87 (2):110-118 | 1985 |
| Besag VE | Bullies and victims in schools: A guide to understanding and management. Library of congress cataloging-publication data.: Open University Press Milton Keynes, Philadelphia;1989. p. 227 | 1989 |
| Kenealy P, Frude N, Shaw W. | An evaluation of the psychological and social effects of malocclusion: some implications for dental policy making. Soc Sci Med. 1989;28(6):583-91. | 1989 |
| Bacher M, Koppenburg P, Klosinski G, Dausch-Neumann D. | Oral stereotypes in school children. Oralprophylaxe. 1990 Dec;12(4):160-70 | 1990 |
| Lovius BB, Jones RB, Pospisil OA, Reid D, Slade PD, Wynne TH. | The specific psychosocial effects of orthognathic surgery. J Craniomaxillofac Surg. 1990 Nov;18(8):339-42 | 1990 |
| Fowkes FG, Fulton PM | Critical appraisal of published research: Introductory guidelines. BMJ 1991;302(6785):1136-1140 | 1991 |
| Boulton MJ, Underwood K. | Bully/victim problems among middle school children. The British journal of educational psychology , 1992; 62 (1):73-87. | 1992 |
| Albino JE, Lawrence SD, Tedesco LA | Psychological and social effects of orthodontic treatment. J Behav Med. 1994 Feb;17(1):81-98 | 1994 |
| Olweus D | Victimisation by peers: Antecedents and long-term outcomes. In Rubin K.H. (Ed.) Asendorf J.B.Social withdrawal, inhibition and shyness in childhood; 1993. p. 315–341 | 1993 |
| Olweus D | Bullying at school: What we know and what we can do: United Kingdom, Blackwell Publishing; 1993. p. 152) | 1993 |
| Olweus D | Bullying at school: Basic facts and effects of a school based intervention program. Journal of child psychology and psychiatry and allied disciplines, 35:1171-1190. | 1994 |
| Turner SR, Thomas PW, Dowell T, Rumsey N, Sandy JR. | Psychological outcomes amongst cleft patients and their families.Br J Plast Surg. 1997 Jan;50(1):1-9. | 1997 |
| Bernstein JY, Watson MW | Children who are targets of bullying: A victim pattern. Journal of Interpersonal Violence 1997;12(1):483–498 | 1997 |
| Phillips C, Bennett ME, Broder HL. | Dentofacial disharmony: psychological status of patients seeking treatment consultation. Angle Orthod. 1998 Dec;68(6):547-56. | 1998 |
| Hawker DS, Boulton MJ | Twenty years' research on peer victimization and psychosocial maladjustment: A meta-analytic review of cross-sectional studies. Journal of Child Psychology and Psychiatry, and allied disciplines 2000;41(4):441-455 | 2000 |
| DiBiase AT, Sandler PJ | Malocclusion, orthodontics and bullying. Dental update 2001;28(9):464-466 | 2001 |
| Carney AG, Merrel KW. | Perspectives on understanding and preventing an international problem. School Psychology International 2001;22(3):364-382. | 2001 |
| Ross DM | Childhood bullying, teasing, and violence: What school personnel, other professionals, and parents can do. 2^nd^ ed. Alexandria, Va.: American Counseling Association; 2003. p. 293) | 2003 |
| Onyeaso CO, Utomi IL, Ibekwe TS. | Emotional effects of malocclusion in Nigerian orthodontic patients. J Contemp Dent Pract. 2005 Feb 15;6(1):64-73. | 2005 |
| Carroll P, Shute R. | School peer victimization of young people with craniofacial conditions: A comparative study, Psychology, Health & Medicine, 2005,10:3, 291-305 | 2005 |
| Marques LS, Barbosa CC, Ramos-Jorge ML, Pordeus IA, Paiva SM | Prevalência de maloclusão e necessidade de tratamento ortodôntico em escolares de 10 a 14 anos de idade em Belo Horizonte, Minas Gerais. Brasil enfoque psicossocial. Cadernos de Saúde Pública. Rio de Janeiro, 21 (4): 1099-1106, jul-ago 2005 | 2005 |
| Rwakatema DS, Ng'ang'a PM, Kemoli AM | Awareness and concern about malocclusion among 12-15 year-old children in Moshi, Tanzania. East African Medical Journal 2006; 83(4):92-97. | 2006 |
| Cheung LK, Loh JS, Ho SM. | Psychological profile of Chinese with cleft lip and palate deformities. Cleft Palate Craniofac J. 2007 Jan;44(1):79-86 | 2007 |
| Vig K. | Quality of Life of Schoolchildren May Be Affected by Poor Alignment of their Teeth. Journal of Evidence Based Dental Practice. Volume 7, Issue 1, March 2007, Pages 35-37 | 2007 |
| Strauss RP, Ramsey BL, Edwards TC, Topolski TD, Kapp-Simon KA, Thomas CR, Fenson C, Patrick DL. | Stigma experiences in youth with facial differences: a multi-site study of adolescents and their mothers. Orthod Craniofac Res. 2007 May;10(2):96-103. | 2007 |
| Bernabé E, de Oliveira CM, Sheiham A. | Condition-specific sociodental impacts attributed to different anterior occlusal traits in Brazilian adolescents. Eur J Oral Sci. 2007 Dec;115(6):473-8. | 2007 |
| Solberg ME, Olweus D, Endresen IM. | Bullies and victims at school: Are they the same pupils? The British journal of educational psychology 2007; 77(2):441-464 | 2007 |
| Fleming PS, Proczek K, DiBiase AT. | I want braces: factors motivating patients and their parents to seek orthodontic treatment. Community Dent Health. 2008 Sep;25(3):166-9. | 2008 |
| Kolawole KA, Otuyemi OD, Adeosun OD | Nicknames and name calling among a population of nigerian schoolchildren. European journal of paediatric dentistry 2009;10(3):115-120 | 2009 |
| Marques LS, Filogônio CA, Filogônio CB, Pereira LJ, Pordeus IA, Paiva SM, Ramos-Jorge ML | Aesthetic impact of malocclusion in the daily living of brazilian adolescents. J Orthod. 2009 Sep;36(3):152-9. | 2009 |
| Moher D, Liberati A, Tetzlaff J, Altman DG, Group P | Preferred reporting items for systematic reviews and meta-analyses: The prisma statement. Journal of clinical epidemiology 2009; 62(10):1006-1012 | 2009 |
| Kolawole K.A.; Otuyemi O.D.; Adeosun O.D | Nicknames and name calling among a population of nigerian schoolchildren. European journal of paediatric dentistry : official journal of European Academy of Paediatric Dentistry. 2009;10: 115-120 | 2009 |
| Craig W, Harel-Fisch Y, Fogel-Grinvald H, Dostaler S, Hetland J, Simons-Morton B | A cross-national profile of bullying and victimization among adolescents in 40 countries. International Journal of Public Health 2009; 54(2):216-224 | 2009 |
| Mandall N, DiBiase A, Littlewood S, Nute S, Stivaros N, McDowall R | Is early class iii protraction facemask treatment effective? A multicentre, randomized, controlled trial: 15-month follow-up. Journal of Orthodontics 2010;37(3);149-161. | 2010 |
| Ometeso BA. | Bullying behaviour, its associated factors and psychological effects among secondary students in Nigeria. The Journal of International Social Research 2010;3:498–509. | 2010 |
| Jung MH. | Evaluation of the effects of malocclusion and orthodontic treatment on self-esteem in an adolescent population. American Journal of Orthodontics and Dentofacial Orthopedics 2010;138(2):160-166. | 2010 |
| Badran, S.A | The effect of malocclusion and self-perceived aesthetics on the self-esteem of a sample of jordanian adolescents. European Journal of Orthodontics 2010;32(6):638-644 | 2010 |
| Verdecchia F, Bee M, Lombardo L, Sgarbanti C, Gracco A. | Influence of anterior tooth alignment on peer perception in 8 to 10 years old children. Eur J Orthod. 2011 Apr;33(2):155-60. doi: 10.1093/ejo/cjq049. Epub 2010 Jul 27. | 2011 |
| Hinggs J, Green S | Cochrane handbook for systematic reviews of interventions. Oxford: The Cochrane collaboration; 2011 | 2011 |
| Seehra J, Fleming PS, Newton T, DiBiase AT. | Bullying in orthodontic patients and its relationship to malocclusion,self-esteem and oral health-related quality of life. Journal of orthodontics 2011;38(4):247-256 (a) | 2011 |
| Seehra J, Newton JT, DiBiase AT | Bullying in schoolchildren - its relationship to dental appearance and psychosocial implications: An update for gdps. British Dental Journal 2011;10(9):411-415 (b) | 2011 |
| De Baets E, Lambrechts H, Lemiere J, Diya L, Willems G. | Impact of self-esteem on the relationship between orthodontic treatment need and oral health-related quality of life in 11- to 16-year-old children. Eur J Orthod. 2012 Dec;34(6):731-7. doi: 10.1093/ejo/cjr088. Epub 2011 Jul 12. | 2012 |
| Kvist T, Annerback EM, Sahlqvist L, Flodmark O, Dahllof G. | Association between adolescents' self-perceived oral health and self-reported experiences of abuse. Eur J Oral Sci. 2013 Dec;121(6):594-9. doi: 10.1111/eos.12084. Epub 2013 Sep | 2013 |
| Al-Bitar Z.B.; Al-Omari I.K.; Sonbol H.N.; Al-Ahmad H.T.; Cunningham S.J | Bullying among jordanian schoolchildren, its effects on school performance, and the contribution of general physical and dentofacial features. American Journal of Orthodontics and Dentofacial Orthopedics: official publication of the American Association of Orthodontists, its constituent societies, and the American Board of Orthodontics. 2013;144: 872-878 | 2013 |
| Brosens V.; Ghijselings I.; Lemiere J.; Fieuws S.; Clijmans M.; Willems G | Changes in oral health-related quality of life reports in children during orthodontic treatment and the possible role of self-esteem: A follow- up study. European Journal of Orthodontics. 2014;36: 186-191 | 2014 |
| Seehra J, Newton JT, Dibiase AT | Interceptive orthodontic treatment in bullied adolescents and its impact on self-esteem and oral-health-related quality of life. European Journal of Orthodontics 2013; 35(5):615-621 | 2013 |
| Al-Bitar ZB, Al-Omari IK, Sonbol HN, Al-Ahmad HT, Cunningham SJ | Bullying among jordanian schoolchildren, its effects on school performance, and the contribution of general physical and dentofacial features. American Journal of Orthodontics and Dentofacial Orthopedics 2013;144(6):872-878 | 2013 |
| Agel M, Marcenes W, Stansfeld SA, Bernabe E | School bullying and traumatic dental injuries in east london adolescents. British Dental Journal 2014;217(12):E26 | 2014 |
| Perillo L, Esposito M, Caprioglio A, Attanasio S, Santini AC, Carotenuto M. | Orthodontic treatment need for adolescents in the Campania region: the malocclusion impact on self-concept. Patient Prefer Adherence. 2014 Mar 19;8:353-9. | 2014 |
| Scheffel DL, Jeremias F, Fragelli CM, Dos Santos-Pinto LA, Hebling J, de Oliveira OBJr. | Esthetic dental anomalies as motive for bullying in schoolchildren. European journal of dentistry 2014;8(1):124-128 | 2014 |
| Pithon MM, Andrade D, Fernandes I, Mendes J, Nunes K, Michele L | Influence of malocclusion on social perceptions of adolescents at public and private schools. European archives of paediatric dentistry 2014;15(1):37-43 | 2014 |
| Malta DC, Porto DL, Crespo CD, Silva MM, de Andrade SS, de Mello FC. | Bullying in brazilian school children: Analysis of the national adolescent school-based health survey (pense 2012). Brazilian journal of epidemiology 2014;17(1):92-105. | 2014 |
| Agel M.; Marcenes W.; Stansfeld S.A.; Bernabe E | School bullying and traumatic dental injuries in east london adolescents. British Dental Journal. 2014;217: E26 | 2014 |
| Serra-Negra JM, Paiva SM, Bendo CB, Fulgencio LB, Lage CF, Correa-Faria P | Verbal school bullying and life satisfaction among brazilian adolescents: Profiles of the aggressor and the victim. Comprehensive Psychiatry 2015; 57:132-139 | 2015 |
| Dorcas OF | Bullying in nigerian secondary schools : Strategies for counseling intervention. Education  Research and  Reviews 2015;10(4): 435–443 | 2015 |
| Dimberg L, Arnrup K, Bondemark L | The impact of malocclusion on the quality of life among children and adolescents: A systematic review of quantitative studies. European journal of orthodontics 2015;37(3):238-247 | 2015 |
| Taibah SM, Al-Hummayani FM | Effect of malocclusion on the self-esteem of adolescents. Journal of Orthodontic Science 2017; 6(4):123-128 | 2017 |
| Kaur P, Singh S, Mathur A, Makkar DK, Aggarwal VP, Batra, M | Impact of dental disorders and its influence on self-esteem levels among adolescents. Journal of Clinical and Diagnostic Research 2017;11(4):05-08 | 2017 |
| Haas MF, Bellato A, Alves GG, Arossi G | Bullying na escola e fatores associados a saúde oral. Adolesc Saude. 2017;14(4):85-96 | 2017 |
| Chikaodi O, Abdulmanan Y, Emmanuel AT, Muhammad J, Mohammed, MA, Izegboya A | Bullying, its effects on attitude towards class attendance and the contribution of physical and dentofacial features among adolescents in northern Nigeria. International Journal of Adolescent Medicine and Health 2017;31(2) | 2017 |
| Taibah S.M.; Al-Hummayani F.M | Effect of malocclusion on the self-  esteem of adolescents. Journal of Orthodontic Science. 2017;6: 123-128 | 2017 |
| Kaur P.; Singh S.; Mathur A.; Makkar D.K.; Aggarwal V.P.; Batra M | Impact of dental disorders and its influence on self esteem levels among adolescents. Journal of Clinical and Diagnostic Research : JCDR. 2017;11: 05-08 | 2017 |
| Shen L, He F, Zhang C, Jiang H, Wang J | Prevalence of malocclusion in primary dentition in Mainland, China, 1988-2017: A systematic review and meta-analysis. Scientific reports 2018;8(1): 4716 | 2018 |
| Eslamipour F, Afshari Z, Najimi A | Prevalence of malocclusion in permanent dentition of iranian population: A review article. Iranian Journal of Public Health 2018;47(2):178-187 | 2018 |
| Julca-Ching K, Carruitero MJ | Impact of the need for orthodontic treatment on academic performance, self-esteem and bullying in schoolchildren. J Oral Res 2019;8(2):99-103 | 2019 |
